# Aurora kinase inhibitors synergize with paclitaxel to induce apoptosis in ovarian cancer cells

**DOI:** 10.1186/1479-5876-6-79

**Published:** 2008-12-11

**Authors:** Christopher D Scharer, Noelani Laycock, Adeboye O Osunkoya, Sanjay Logani, John F McDonald, Benedict B Benigno, Carlos S Moreno

**Affiliations:** 1 Department of Pathology & Laboratory Medicine, Emory University School of Medicine, Atlanta, GA 30322, USA; 2 Program in Genetics & Molecular Biology, Emory University, Atlanta, GA, USA; 3 School of Biology, Georgia Institute of Technology, Atlanta, GA 30332, USA; 4 Ovarian Cancer Institute, Atlanta, GA 30342, USA; 5 Winship Cancer Institute, Emory University School of Medicine, Atlanta, GA 30322, USA

## Abstract

**Background:**

A large percentage of patients with recurrent ovarian cancer develop resistance to the taxane class of chemotherapeutics. While mechanisms of resistance are being discovered, novel treatment options and a better understanding of disease resistance are sorely needed. The mitotic kinase Aurora-A directly regulates cellular processes targeted by the taxanes and is overexpressed in several malignancies, including ovarian cancer. Recent data has shown that overexpression of Aurora-A can confer resistance to the taxane paclitaxel.

**Methods:**

We used expression profiling of ovarian tumor samples to determine the most significantly overexpressed genes. In this study we sought to determine if chemical inhibition of the Aurora kinase family using VE-465 could synergize with paclitaxel to induce apoptosis in paclitaxel-resistant and sensitive ovarian cancer cells.

**Results:**

Aurora-A kinase and TPX2, an activator of Aurora-A, are two of the most significantly overexpressed genes in ovarian carcinomas. We show that inhibition of the Aurora kinases prevents phosphorylation of a mitotic marker and demonstrate a dose-dependent increase of apoptosis in treated ovarian cancer cells. We demonstrate at low doses that are specific to Aurora-A, VE-465 synergizes with paclitaxel to induce 4.5-fold greater apoptosis than paclitaxel alone in 1A9 cells. Higher doses are needed to induce apoptosis in paclitaxel-resistant PTX10 cells.

**Conclusion:**

Our results show that VE-465 is a potent killer of taxane resistant ovarian cancer cells and can synergize with paclitaxel at low doses. These data suggest patients whose tumors exhibit high Aurora-A expression may benefit from a combination therapy of taxanes and Aurora-A inhibition.

## Background

Eukaryotic cells have developed stringent cell cycle controls to ensure mitosis occurs consistently error free. Cell cycle checkpoints have evolved to ensure the inheritance of undamaged DNA, and that each daughter cell receives the correct complement of chromosomes. Aberrant expression and function of proteins that regulate the mitotic spindle, and other cell cycle checkpoints can lead to aneuploidy and contribute to cancer progression [1]. The Aurora family of evolutionarily conserved serine/threonine kinases regulates entry into mitosis, centrosome maturation and the mitotic spindle checkpoint [2]. Mammalian genomes contain three members of this kinase family, Aurora-A, B and C. Aurora-A was first characterized in *Drosophila melanogaster *where mutants exhibited defects in centrosome separation [3]. Aurora-B is a chromosomal passenger protein that begins mitosis localized to the centromeres but at the onset of anaphase relocates to the spindle equator [4]. Aurora-B kinase is known to regulate processes such as kinetochore and microtubule interactions [5-8] and cytokinesis [9,10]. Aurora-C is expressed specifically in the male testis [11] and has meiotic functions [12].

Aurora-A is critical for mitotic entry, as well as the mitotic spindle checkpoint involving chromosome maturation and segregation [13-15]. Two proteins known to bind and initiate activation of Aurora-A are TPX2 [16,17] and Ajuba [13]. Upon binding, TPX2 or Ajuba stimulate Aurora-A to undergo autophosphorylation and subsequent activation. Once activated, Aurora-A phosphorylates downstream targets such as TPX2, thus regulating the attachment of microtubules to the kinetochore during spindle assembly [18-20]. Aurora-A also phosphorylates the tumor suppressor protein p53, resulting in MDM2 dependent degradation and cell cycle progression [21]. Aurora-A is overexpressed in ovarian [22-24], breast [25], colorectal [26] and metastatic prostate cancer [27] and is upregulated in response to simian virus 40 (SV40) small tumor (ST) antigen [28]. In addition, amplification of human chromosome 20q13.2, which contains Aurora-A, frequently occurs in ovarian cancer [29]. Overexpression of Aurora-A causes transformation in rodent fibroblasts [30] and tumors in nude mice [31], consistent with the possibility that Aurora-A is an oncogene.

The current standard of care for advanced ovarian cancer is debulking surgery followed by combination chemotherapy of carboplatin and paclitaxel [32]. Unfortunately, the majority of patients relapse within 18 months of first-line therapy, and 24–59% of relapse patients treated with paclitaxel progress to resistant disease [33]. Paclitaxel causes cell death by stabilization of microtubule dynamics resulting in activation of the spindle assembly checkpoint and apoptosis [34]. Previous studies have investigated the link between Aurora-A levels and sensitivity or resistance to paclitaxel. One study demonstrated that overexpression of Aurora-A in HeLa cells induces resistance to paclitaxel [35] while another study reported sensitization of pancreatic cancer cells to paclitaxel by siRNA knockdown of Aurora-A [36]. Interestingly, a recent study in ovarian cancer cells reported that overexpression of Aurora-A could increase cell survival in the presence of paclitaxel [37].

Through microarray profiling of ovarian cancer samples, we have observed that Aurora-A was significantly overexpressed in ovarian carcinomas compared to adenomas. We confirmed Aurora-A expression at the protein level by staining tissue microarrays from the same patients. Recently, Aurora kinases have been exploited as novel drug targets with the development of a handful of small molecule inhibitors, all of which have been or are in clinical trials (Reviewed in [38]). To determine if the Aurora kinase family is an effective therapeutic target for ovarian tumors that have acquired resistance to paclitaxel, we tested the ability of VE-465, an Aurora kinase family inhibitor (gift of Merck & Co. and Vertex Pharmaceuticals), to induce apoptosis in the presence and absence of paclitaxel in taxol-sensitive 1A9 and taxol-resistant PTX10 ovarian cancer cells [39]. VE-465 potently induced apoptosis in both paclitaxel resistant and sensitive ovarian cancer cells. In addition, VE-465 synergistically enhanced apoptosis in combination with paclitaxel in taxol-sensitive cells at low doses (1–10 nM). Our data indicate that VE-465 is effective at inducing apoptosis in both taxol-sensitive and taxol-resistant ovarian cancer cell lines, and thus may be an effective therapy for patients with ovarian cancer, including those patients with taxol-resistant disease.

## Methods

### Tumor samples, RNA isolation, Microarray Hybridization and Normalization

A detailed explanation of patient samples and microarray hybridization and normalization techniques is described elsewhere [22]. The complete dataset is available at the NCBI GEO website (, accession number GSE7463) and at the author's website .

### Cell Culture and Drug Treatment

PTX10 and 1A9 cells were cultured in RPMI media (Mediatech, Herndon, VA) supplemented with 10% fetal bovine serum and grown in 5% CO_2 _at 37°C. Two days before treatment 1.5 × 10^5 ^cells were seeded in each well of a 6-well plate (Corning, Corning, NY). On day one of treatment combinations of 15 ng/mL paclitaxel (Sigma-Aldrich, St. Louis, MO) and either Dimethyl Sulfoxide (DMSO) control or the indicated concentration of of VE-465 (Vertex Pharmaceuticals, Abingdon, United Kingdom) were added to 2 mL of fresh RPMI and incubated for 96 hours prior to FACS analysis or caspase 3/7 activity assays.

### Fluorescence Activated Cell Sorting (FACS) Analysis

Following drug treatment, cells were washed from the plate in media, centrifuged at 3000 rpm to pellet and washed once with cold PBS. Pellets were resuspended and fixed in 70% Ethanol/PBS at -20°C overnight. On the day of analysis, pellets were washed once with PBS and digested with 500 μl of 0.1 mg/mL PBS/RNaseA (Sigma-Aldrich, St. Louis, MO) by incubating at 37°C for 15 minutes. DNA content was assessed by staining with 500 μl of 25 μg/mL PBS/Propidium Iodide (Sigma-Aldrich, St. Louis, MO). Cell suspensions were transferred to 5 mL collection tubes for FACS analysis. Samples were processed using a Becton Dickson FACSCalibur analyzer (Becton Dickson, San Jose, CA) and data analyzed using the FlowJo software package (Tree Star, Ashland, OR).

### Drug Treatment and Caspase Assay

One day before drug treatment, each well of a white-walled, 96 well luminometer plate (Nalge Nunc International, Rochester, NY) was coated with a 1:4 dilution of BD matrigel matrix (BD biosciences, Bedford, MA) and RPMI media. The plates were incubated at room temperature for one hour and excess matrigel was removed before 4800 cells were seeded in each well in triplicate. On day one of treatment, cells were treated with or without 15 ng/mL paclitaxel (Sigma-Aldrich, St. Louis, MO) plus varying concentrations and combinations of VE-465 (Vertex Pharmaceuticals, Abingdon, United Kingdom), or with 50 μM z-vad (EMD Chemicals, San Diego, CA). Z-vad is a general caspase inhibitor and was used as a negative control to block caspase activity and apoptosis. Control cells were left untreated. Three independent biological replicates were performed, luminescence measured and data analyzed.

The Caspase-Glo™ 3/7 Assay (Promega, Madison, WI) lyophilized substrate (DEVD-aminoluciferin powder) was resuspended in Caspase- Glo™ 3/7 lysis buffer and equilibrized to room temperature. Forty-eight or 72 hours after cell treatment, the Caspase- Glo™ 3/7 reagent was added in a 1:1 volume ratio to each well of the 96 well luminometer plate. Immediately following the addition of the reagent, the contents of the wells were gently mixed with a plate shaker at 500 rpm for 30 seconds. After one hour incubation, the luminescence was measured with a Synergy HT plate reader (BioTek Instruments, Winooski, VT). Culture medium was used as a blank and "no-cell background" values were determined.

### Immunofluorescence

PTX10 and 1A9 cells were grown on cover slips (Fisher Scientific, Hampton, NH) in 6-well culture dishes (Corning, Corning, NY). Cells were washed 3 times with cold PBS and fixed in 4% paraformaldehyde for 15 minutes at room temperature, permeablized on ice for 2 minutes in 0.5% Tween-20/PBS and blocked in 5% nonfat dry milk (NFDM) for 30 minutes at room temperature. Mitotic cells were stained with anti-phospho-Histone H3 Serine 10 (Upstate, Charlottesville, VA) with 5% NFDM at a 1:200 dilution for 2 hours at 4°C. Secondary antibody of anti-Rabbit AlexaFluor 488 (Molecular Probes, Eugene, OR) was applied at a 1:400 dilution for 45 minutes at room temperature. Cells were washed 3 times in PBS and stained with TOPro (Molecular Probes, Eugene, OR) at a concentration of 3 μg/μl for 15 minutes to reveal the nucleus. Cover slips were mounted on slides and visualized using a Zeiss Axiovert 35 fluorescence microscope.

### Western Blot

60% conflutent cells were lysed in lysis buffer (0.137 M NaCl, 0.02 M TRIS pH 8.0, 10% Glycerol, and 1% NP-40), 50 μg total lysate separated by SDS-PAGE electrophoresis and transferred to nitrocellulose for immunoblotting. Immunoblots were probed with an antibody to Aurora-A (Abcam Inc., Cambridge, MA), Aurora-B (GenScript, Piscitaway, NJ), phosphoAurora-A and -B (Cell Signaling, Danvers, MA), p53 (Santa Cruz Biotechnology, Santa Cruz, CA) and phospho(S315)p53 (Cell Signaling, Danvers, MA). To ensure equal loading blots were then probed with a monoclonal antibody to PP2A, catalytic subunit (BD Biosciences, San Jose, CA).

### Tissue Microarray Analysis

TMA sections were stained at the WCI Tissue and Pathology Core Facility  with H&E and with Aurora A antibody (1:300 dilution, Abcam, Cambridge, MA). Staining was scored on a four level scale (0 = no staining, 1 = weak staining, 2 = moderate staining, 3 = intense staining) by a GU pathologist.

## Results

### Expression Profiling of Ovarian Cancer Patients

**Figure 1:**
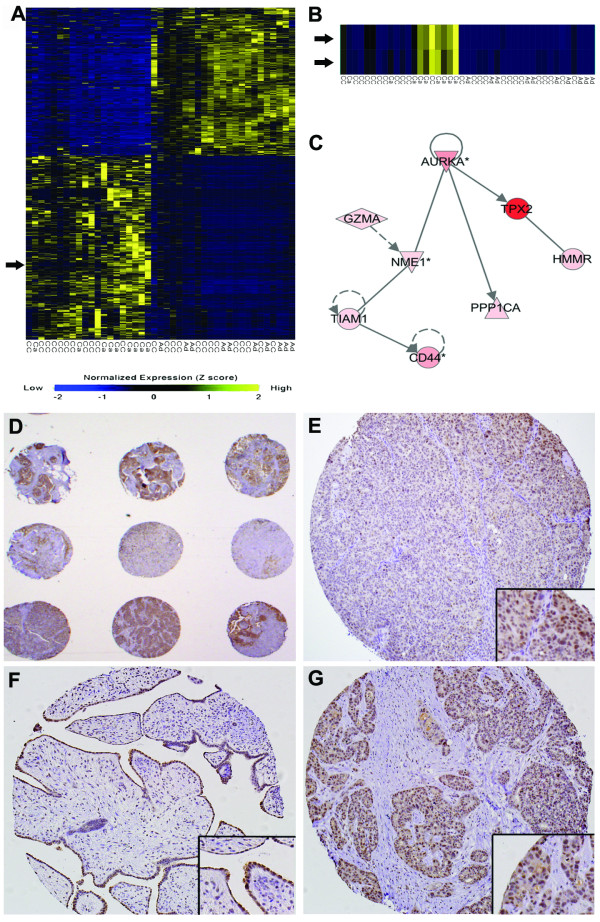
Aurora-A is overexpressed in carcinomas. Heat map image of Z-score normalized microarray expression data from Affymetrix U95A gene chips. Genes with lower expression compared to normal tissue are shown in blue and yellow indicates genes that are overexpressed. **(A) **Heat map representing the entire data set. Arrow indicates Aurora-A. **(B) **Aurora-A is overexpressed 5 fold in carcinomas compared to adenomas. Both Aurora-A probes are shown. Ca – carcinoma, Ad – adenoma, CC – cancers pre-treated with chemotherapy. **(C) **Ingenuity Pathway Assist analysis of significantly overexpressed genes. Diagram represents an interaction network of the 8 genes and Aurora-A kinase. **(D) **Low power (2×) image of ovarian tissue microarray stained for Aurora A by immunohistochemistry. **(E) **Aurora-A staining of TMA core of ovarian carcinoma without adjuvant chemotherapy (20×). **(F) **Aurora-A staining of TMA core of benign ovarian tissue (20×). **(G) **Aurora-A staining of TMA core of ovarian carcinoma with adjuvant chemotherapy (20×).

We sought to establish gene expression profiles of ovarian cancer patients in order to determine genes whose expression was significantly different between carcinoma, adenoma and tumors pretreated with chemotherapy. Expression profiling of 9 carcinoma, 10 adenoma and 24 neoadjuvant chemotherapy-treated ovarian cancer patients was performed using an Affymetrix U95A gene chip, and a comprehensive analysis of these results has been published elsewhere [22]. Significance Analysis of Microarray (SAM) followed by Z-score normalization revealed 962 probe sets significantly upregulated and 565 probe sets significantly down regulated at least two fold (Fig. 1A). Consistent with previous reports [23], we observed Aurora-A to be significantly overexpressed 5-fold in ovarian cancer carcinoma patients compared to adenomas (Fig. 1B). We also observed by SAM analysis Aurora-A to be overexpressed 2.3 fold in carcinomas pretreated with chemotherapy relative to adenomas. SAM analysis did not reveal Aurora-B or C to be significantly over or underexpressed in this dataset. Interestingly, Ingenuity Pathway Assist analysis  of significantly altered genes revealed that seven genes known to interact with Aurora-A were also upregulated at least two-fold (Fig. 1C and Table 1). This network is based on the published interactions [16,40-51] present in the Ingenuity Pathways knowledgebase. Among the most highly expressed is the known Aurora-A activator TPX2 which was overexpressed 15-fold. To confirm these observed changes in gene expression by an independent method, we measured the mRNA levels of Aurora-A, TPX2, and NME-1 by quantitative real-time PCR (qPCR) (Table 2).

**Table 1:** Ingenuity Pathway Assist analysis of genes involved in the Aurora-A kinase pathway. Data represents fold enrichment in carcinoma patients versus adenoma patients. *SAM analysis estimated the False Discovery Rate for all genes to be 0.

*Affymetrix Probe ID*	*Gene*	*Name*	*Fold Change*
39109_at	TPX2	TPX2, microtubule associated, homolog	15.42
1125_s_at; 1126_s_at	CD44	CD44 molecule	4.51
36863_at	HMMR	Hyaluronan-mediated motility receptor	2.73
32157_at	PPP1CA	Protein phosphatase 1, catalytic subunit, alpha isoform	2.46
40757_at	GZMA	Granzyme A	2.26
1985_s_at	NME1	Non-metastatic cells 1	2.24
38370_at	TIAM1	T-cell lymphoma invasion and metastasis	2.18

**Table 2:** Confirmation of increased mRNA by QRT-PCR. RNA from eight patient samples (four carcinoma-like and four adenoma-like) was analyzed by QRT-PCR, confirming increased expression levels measured by microarray analysis.

*Gene*	*Fold Change (qPCR)*	*Fold Change (Microarray)*
TPX2	27.6	15.4
AURKA	1.7	5.1
NME-1	3.0	2.1

### Ovarian Cancer Tissue Microarray Analysis of Aurora-A

To characterize the level of expression of Aurora-A at the protein level in ovarian cancers and benign tissues, we stained two ovarian cancer tissue microarrays (TMAs) with antibody to Aurora-A. The TMAs contained 212 cores from 35 patients (7 benign, 7 carcinoma without chemotherapy, and 21 carcinoma with adjuvant chemotherapy). Each core was scored for intensity of staining (1 = weak, 2 = moderate, 3 = strong), as well as the percentage of total cells positive for Aurora-A, and data averaged for each patient's cores. The TMA staining data, including detailed patient information is summarized in Table 3. On average, the benign tumors contained the highest percentage of cells staining positive for Aurora-A (80% ± 17%) while the carcinomas displayed a lower percentage of cells with positive staining (61% ± 22%) (Table 3). Patients with neoadjuvant therapy displayed an intermediate percentage of cells staining positive for Aurora-A (73% + 15%), but these differences were not statistically significant with this small a patient sample. While the overall number of cells that stained positive for Aurora-A were higher in the carcinomas due to increased epithelial content, the intensity of the staining was equivalent with benign ovarian epithelial cells (Fig. 1D–G). Average staining intensities were 2.5 ± 0.5 for benign tissues, 2.2 ± 0.6 for carcinomas with adjuvant chemotherapy, and 2.1 ± 0.5 for carcinomas without adjuvant chemotherapy. Thus, the higher mRNA signal for Aurora-A in ovarian cancers is likely due to the fact that there is much higher epithelial than stromal content in these tissues compared to benign tissues (compare Figs. 1E and 1F). Nevertheless, the ovarian cancer cells could be more sensitive to inhibition of Aurora A than normal cells, and thus determination of the optimal dose of Aurora A inhibitors will be critical for optimizing treatment regimens.

**Table 3:** Summary of staining and detailed patient data for the ovarian tumor tissue microarray stained with anti-Aurora-A antibody.

**Tumor Type**	**Stage**	**Grade**	**No. of Patients**	**Age at Surgery**	**Survival (Months)**	**TMA Score**	**% Cells Aurora-A Positive**
**Benign**	-	-	7	65 (10)	-	2.5 (0.5)	80 (17)

**Carcinoma No Chemotherapy**	I	3	1	47	-	2.9	84
	
	II	3	1	61	-	2	44
	
	III	2	1	45	-	2.4	75
		
		3	3	61 (14)	-	2.4 (0.7)	65 (20)
	
	IV	3	1	74	-	1.3	30

**Carcinoma With Chemotherapy**	III	1	1	55	53	1.8	59
		
		2	9	63 (13)	29 (16)	2 (0.6)	70 (15)
		
		3	9	61 (8)	33 (6)	2.3 (0.5)	79 (14)
	
	IV	2	1	51	62	1.8	48
	
		3	1	72	22	2	78

Brackets represent standard deviations.

### Aurora Kinases are expressed in Ovarian Cancer Cell lines

**Figure 2:**
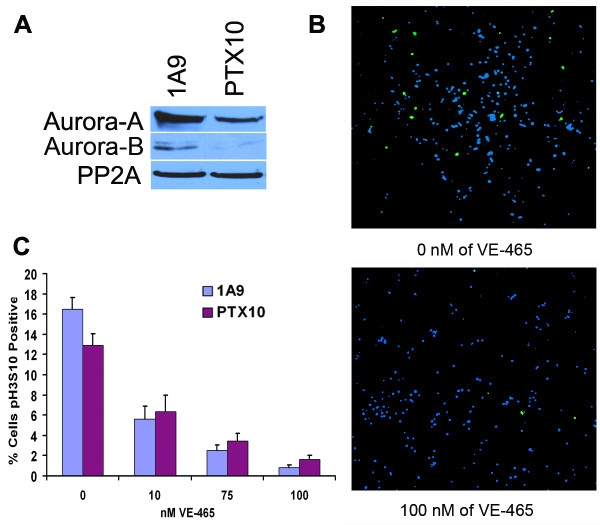
VE-465 inhibits the Aurora kinases. **(A) **Immunoblot analysis of whole cell lysates from 1A9 and PTX10 cell lines probed for Aurora-A, Aurora-B and PP2A as a loading control. **(B) **Paclitaxel-resistant PTX10 and IA9 cells were treated for 48 hours with VE-465. Following treatment, mitotic cells were assessed by staining for Histone H3 phosphorylated on Ser10 (pH3S10), a marker of mitosis and an Aurora-B substrate (green). Nuclear chromatin was visualized with the To-Pro (blue) counter stain to indicate total number of cells. **(C) **Ten random fields were sampled for each concentration and percentage of pH3S10 positive cells calculated.

It has been previously shown that overexpression of Aurora-A can induce resistance to paclitaxel in a cell culture model [35]. To assess the effect of Aurora kinase inhibition on taxol-sensitive and taxol-resistant ovarian cell lines, we examined taxol-sensitive 1A9 cells, and taxol-resistant PTX10 cells that are derived from the 1A9 cell line [39]. Unfortunately, the mechanism of taxol resistance in PTX10 is not by Aurora-A overexpression. Rather, PTX10 cells harbor a point mutation in the M40 β-tubulin isotype resulting in a phenylalanine to valine mutation [39] that is hypothesized to alter the binding of paclitaxel to microtubules. In fact, 1A9 cells express a roughly two-fold higher level of Aurora-A, than PTX10 cells as determined by western blot (Fig. 2A), and 1A9 cells demonstrated low levels of Aurora-B expression whereas Aurora-B was barely detectable in the PTX10 cell line. Thus, it was not known whether Aurora-kinase inhibition would alter the effect of paclitaxel, or induce apoptosis via other mechanisms. Consequently, we proceeded to test both taxol-sensitive 1A9 cells and taxol-resistant PTX10 cells.

### VE-465 Inhibits the Aurora Kinases

We obtained an Aurora kinase inhibitor VE-465 (gift of Merck & Co., West Point, PA and Vertex Pharmaceuticals, Oxford, UK). VE-465 has a slightly higher K_i _than VX-680, but is still highly specific for the three kinases (Aurora-A K_i _= 1 nM, Aurora-B K_i _= 26 nM, Aurora-C K_i _= 9 nM, FLT-3 K_i _= 29 nM, Abl K_i _= 44 nM) (data from Merck & Co). VE-465 has been shown to have some activity against mutant BCR-ABL kinase in mice at 75 mg/kg [52] and to induce apoptosis in multiple myeloma cells at 100–500 nM [53]. Serine 10 on Histone H3 is a highly conserved residue and is phosphorylated by Aurora-B kinase upon entry into mitosis [54,55]. We used immunocytochemistry to determine the percentage of cells positive for histone H3 phosphorylated on Serine 10 (pH3S10) after treatment with VE-465. Treatment with 100 nM of VE-465 caused significant decrease in pH3S10 positive cells, whereas a DMSO control treatment had no effect (Fig. 2B). Quantification of 10 random fields indicated a decrease of 7.9 fold in PTX10 and 20.9 fold in 1A9 mitotic cells when treated with 100 nM of VE-465 (Fig. 2C). These results demonstrate that VE-465 effectively inhibits Aurora B kinase in a dose dependent manner and prevents the phosphorylation of a known mitotic marker in ovarian cancer cells.

### VE-465 Induces Apoptosis in Ovarian Cells

**Figure 3:**
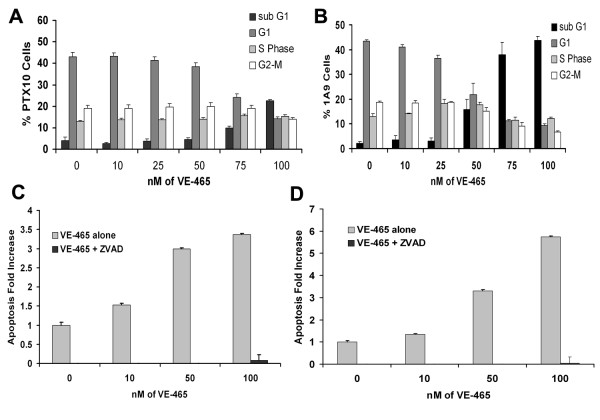
Inhibition of Aurora kinases results in cell death. Cells were treated for 96 hours with differing doses of VE-465. **(A) **PTX10 cells **(B) **1A9 cells. Following treatment cells were harvested, fixed and stained with propidium iodide before analysis by Flow Cytometry. The sub G0/G1 population represents apoptotic cells. Each time point represents data from at least 3 independent experiments. Caspase 3/7 assays of PTX10 **(C) **and 1A9 **(D) **cells treated with increasing doses of VE-465 demonstrate dose-dependent increase in apoptosis. The caspase activity was blocked by the pan-caspase inhibitor Z-VAD.

We hypothesized that treatment with VE-465 would induce apoptosis due to misregulation of the cell cycle or because of the polyploid nature of cells that did manage to complete mitosis. We treated 1A9 and PTX10 cells with DMSO (control) or 10, 25, 50, 75 and 100 nM of VE-465 for 96 hours and examined DNA content by propidium iodide staining followed by flow cytometry. Fragmented DNA was measured as a sub G0/G1 peak and was analyzed as a measure of apoptosis. After 96 hours, cell death in the parental 1A9 cell line was increased from 2.15% to 43.6% (Fig. 3B) and from 4.2% to 22.6% (Fig. 3A) in the paclitaxel resistant PTX10 cell line, a roughly 5-fold increase. It is also important to note that as the concentrations of VE-465 increased, both cell lines became increasingly aneuploid (data not shown). After 96 hours there were clearly cells with an array of DNA content ranging from 4 n to 10 n, suggesting that many ovarian cancer cells treated with VE-465 bypass the spindle checkpoint, producing errors in chromosomal segregation.

Consistent with the higher level of expression of Aurora-A, and especially Aurora-B, the 1A9 cells (Figure 2A), were more sensitive than PTX10 cells to VE-465 inhibition treatment at doses of 50, 75, or 100 nM (compare Figures 3A and 3B).

To further confirm that the sub G0/G1 peak was due to apoptosis and not necrosis, we performed Caspase 3/7 assays using a luminescent detection method. Treatment of 1A9 and PTX10 cells with VE-465 resulted in a dose-dependent increase in Caspase 3 and Caspase 7 activity that was inhibited by pretreatment with the general caspase inhibitor Z-VAD (Fig. 3C and 3D).

### VE-465 Promotes Apoptosis in a Paclitaxel Resistant Cell Line at high doses

**Figure 4:**
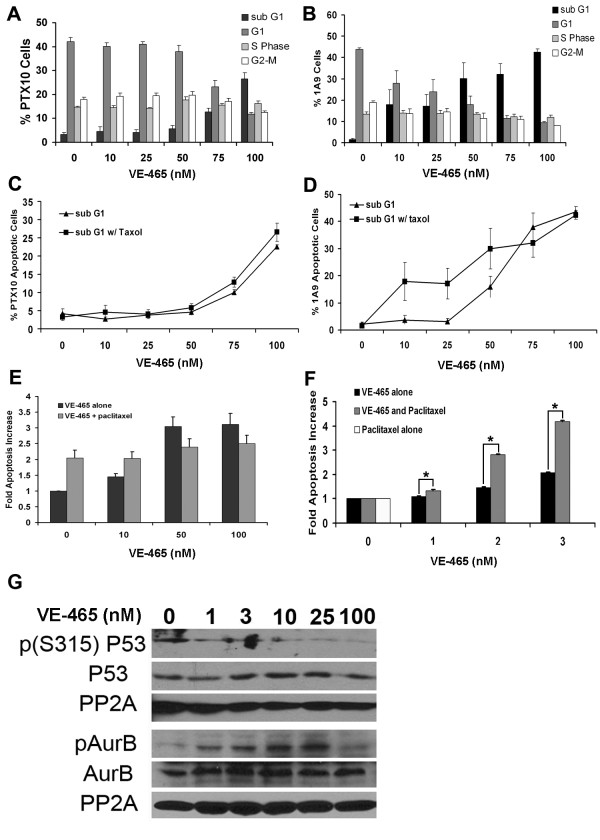
VE-465 induces cell death in the presence of paclitaxel. Cells were treated for 96 hours with differing doses of VE-465 in the presence of 15 ng/mL paclitaxel. **(A) **PTX10 cells **(B) **1A9 cells. Analysis was performed as described in Figure 3. The sub G0/G1 population represents apoptotic cells. Each time point represents data from at least 3 independent experiments. Paclitaxel and VE-465 did not synergize to cause apoptosis in PTX10 **(C) **or 1A9 **(D) **cells. Percent of apoptotic cells are plotted for cells treated for 96 hrs with VE-465 alone or VE-465 and 15 ng/mL paclitaxel. Triangles – cells treated with increasing concentrations of VE-465. Squares – cells treated with increasing concentrations of VE-465 in the presence of 15 ng/mL paclitaxel. **(E) **Caspase 3/7 assays of PTX10 cells treated with 10–100 nM of VE-465 alone or in combination with 15 ng/mL paclitaxel. Confirming flow cytometry data, combination treatment with paclitaxel and VE-465 did not synergistically increase apoptosis in the PTX10 cell line. **(F) **Caspase 3/7 assays of 1A9 cells treated with 1–3 nM of VE-465 alone, 15 ng/mL paclitaxel alone, or in combination with 15 ng/mL paclitaxel. A dose of 3 nM VE-465 alone induced 2-fold more apoptosis than 15 ng/mL paclitaxel, whereas combined 3 nM VE-465 and 15 ng/mL paclitaxel synergistically induced 4.5-fold more apoptosis than 15 ng/mL paclitaxel alone. (* = p-value less than 0.0025 by students T-test.) **(G) **Immunoblot of 1A9 cells treated with increasing concentrations of VE-465 for 96 hours. The kinase activity of Aurora-A and Aurora-B is suppressed in a dose-dependent manner consistent with the known K_i _values of VE-465. Phosphorylation of the Aurora-A target p53 (S315) is inhibited at doses of 1 nM and higher whereas auto-phosphorylation of Aurora-B (T232) is only inhibited at doses exceeding 25 nM.

To determine if VE-465 could induce apoptosis in the presence of paclitaxel, we treated 1A9 and PTX10 cells with DMSO (control) and 10, 25, 50, 75, and 100 nM of VE-465 in the presence of 15 ng/mL paclitaxel for 96 hours. In the parental 1A9 cell line, paclitaxel alone caused a slight increase in apoptotic cells, and the addition of VE-465 significantly increased the number of sub G0/G1 cells (Fig. 4B). Consistent with their phenotype [39], PTX10 cells were resistant and proliferated in the presence of 15 ng/mL paclitaxel. The PTX10 cell line exhibited little cell death in low doses of VE-465, but as the concentrations approached 100 nM the percentage of apoptotic cells increased 8-fold (Fig. 4A). The presence of both drugs, paclitaxel and VE-465, did not act synergistically in the PTX10 or 1A9 cell lines at high concentrations as the levels of cell death were only slightly increased when treated with VE-465 in the presence of paclitaxel (Fig. 4C and 4D). Caspase 3/7 assays of PTX10 cells confirmed that there was no statistically significant difference in apoptosis induction between cells treated with VE-465 alone or in combination with 15 ng/mL paclitaxel (Fig. 4E).

### VE-465 Synergizes with paclitaxel to induce apoptosis at low doses specific to Aurora-A

We observed increased apoptosis at low doses of VE-465 in combination with 15 ng/mL paclitaxel in the paclitaxel-sensitive 1A9 cells (Fig. 4C). Therefore, we tested if doses of VE-465 that were specific to Aurora-A (3 nM or less) could synergize with paclitaxel to induce apoptosis in the 1A9 cell line. VE-465 alone induced 2-fold more apoptosis than 15 ng/mL paclitaxel alone (Fig. 4F). Compared to 15 ng/mL paclitaxel alone, 3 nM VE-465 combined with 15 ng/mL paclitaxel to cause a roughly 4.5-fold increase in cell death as measured by caspase 3/7 activity assay (Fig. 4F). To confirm the effects were due to Aurora-A specific inhibition, we treated 1A9 cells with both low and high doses of VE-465 for 96 hours and probed immunoblots for phospho-Aurora-B (T232) and phospho-p53 (S315) (Fig. 4G). p53(S315) is phosphorylated by Aurora-A but not Aurora-B [21]. Aurora B auto-phosphorylates threonine residue 232 (T232) upon activation [56]. Following VE-465 treatment, phoshpo-p53 levels are reduced at doses of 1 nM and higher, indicating an inhibition of Aurora-A activity. As expected, Aurora-B kinase activity was inhibited only at doses of VE-465 that exceeded 25 nM. The level of inhibition we observed is in agreement with the K_i _values for Aurora-A (1 nM) and Aurora-B (25 nM), respectively. These results show that VE-465 by itself can induce apoptosis, and can synergize with paclitaxel at Aurora-A specific concentrations (< 5 nM) to enhance cell killing.

## Discussion

Recently, we identified Aurora-A kinase to be significantly overexpressed in carcinoma patients compared to adenomas [22]. Our data suggested that reduced p53 activity can lead to improved clinical outcome for ovarian cancer patients undergoing chemotherapy [22]. One mechanism that might contribute to this phenomenon is that Aurora-A renders cells resistant to paclitaxel-induced apoptosis and stimulates Akt1 and Akt2 activity in wild-type p53 but not p53-null ovarian cancer cells [37]. Thus, p53-null tumors would be more responsive to chemotherapy regimens. Here, we have shown that the mitotic kinase Aurora-A is overexpressed in ovarian carcinomas compared to adenomas. Furthermore, we have demonstrated that the pan-Aurora inhibitor VE-465 can synergize with paclitaxel to induce apoptosis and is a potent killer of taxane-sensitive and resistant ovarian cancer cells.

Although other Aurora family members were not overexpressed, other genes known to interact with Aurora-A kinase were significantly increased. One of the most significantly overexpressed was TPX2, an activator and substrate of Aurora-A [16,17]. Recently, a link between another Aurora-A substrate, BRCA1, and TPX2 has been demonstrated [57]. Juokov et al. showed that loss of BRCA1 expression leads to mislocalization of TPX2 along microtubules instead of at the aster poles, suggesting a mechanism by which BRCA1 mutation could lead to chromosomal instability [57]. TPX2 was overexpressed 15-fold in carcinomas and provides a possible mechanism for increased activation of Aurora-A kinase. These observations have implications for ovarian cancer because overexpression of Aurora-A can induce resistance to the chemotherapeutic paclitaxel [35]. We predicted that ovarian cancer patients who overexpress Aurora-A would have a higher chance of becoming resistant to taxanes and possibly benefit from a different treatment strategy targeted at Aurora-A and other Aurora family members. To test this prediction, we evaluated the compound VE-465 as a pan-Aurora kinase inhibitor and inducer of apoptosis in ovarian cancer cell lines. Although VE-465 is not specific to Aurora-A, it is highly selective and effective at inhibiting Aurora family kinases and offered a unique opportunity to evaluate the entire family of kinases as a therapeutic target. Our results indicate that VE-465 is able to induce apoptosis in the paclitaxel resistant, ovarian cancer cell line PTX10 in a dose dependent manner and synergize with paclitaxel in the 1A9 paclitaxel-sensitive cell line.

VE-465 and paclitaxel are both drugs that function by targeting mitotic cells, but induce apoptosis by different mechanisms. Paclitaxel alters microtubule dynamics and induces the spindle checkpoint resulting in mitotic arrest and eventual apoptosis. VE-465, on the other hand, inhibits the activity of the Aurora kinase family and subsequent mitotic entry. We found that many PTX10 cells treated with VE-465 bypass the spindle checkpoint resulting in missegregation of chromosomes and aneuploidy, possibly due to the inhibition of other family members such as Aurora-B. Thus, in addition to inhibiting mitotic entry, VE-465 appears to induce apoptosis by causing catastrophic chromosomal abnormalities due to the absence of an intact spindle assembly checkpoint in cells that do proceed through mitosis.

Intriguingly, 1A9 cells were more sensitive to VE-465 than PTX10 cells and this correlates with the roughly two fold higher expression of Aurora-A in the 1A9 cell line. Significant cell death was observed at low concentrations in 1A9 cells such as 1–25 nM relative to 50–75 nM for PTX10 cells, suggesting that at low doses VE-465 synergizes with paclitaxel in taxol-sensitive ovarian cancer cells. Interestingly, at low concentrations VE-465 has a K_i _more specific to Aurora-A (1 nM) than Aurora-B (26 nM) or -C (9 nM). This suggests the synergistic effects are due to the specific inhibition of Aurora-A and not other family members. However, at higher concentrations, we found no evidence that paclitaxel and VE-465 synergized to induce apoptosis in PTX10 cells. This could be because a very high percentage of cells are undergoing apoptosis at high doses, or possibly due to the inherent nature of the resistance of PTX0 cells. PTX10 cells harbor a point mutation in the M40 β-tubulin isotype resulting in a phenylalanine to valine mutation [39] which may alter the binding of paclitaxel to microtubules. It is possible that this particular form of resistance does not coincide with the function of Aurora kinases and therefore no synergism is seen when treating with a combination of both drugs. Tumors that exhibit other forms of taxane resistance such as Aurora-A overexpression, alternate point mutations, modulations in tubulin isotypes, decreased tubulin expression and changes in post-translational modifications may respond synergistically when treated with VE-465 and paclitaxel. Alternatively, a synergistic effect may be observed prior to the acquisition of taxol resistance, or in combination with other drugs that target different cellular pathways such as tyrosine kinase receptor signals or apoptosis resistance pathways. Aurora kinase inhibitors represent a promising alternative to taxane therapy, especially for patients who overexpress the mitotic kinase Aurora-A, or other family members, or whose disease continues to progress during taxane therapy [58].

Treatment of patients with different drugs in a serial fashion allows for clones that are resistant to one therapy to arise by drug-resistance selection. However, combinatorial therapies may be more effective, as has been shown using cocktail therapies for the treatment of the rapidly evolving human immunodeficiency virus [59]. Thus, initial combinatorial chemotherapy using Aurora-inhibitors, paclitaxel, and other chemotherapeutic agents could be an effective approach to prevent the development of chemo-resistant ovarian cancers.

## Conclusion

In summary, we have shown the mitotic kinase Aurora-A to be overexpressed in ovarian carcinomas compared to adenomas. Furthermore, we demonstrated the pan-Aurora inhibitor VE-465 can synergize with paclitaxel to induce apoptosis and is a potent killer of taxane-sensitive and resistant ovarian cancer cells. Our results suggest that Aurora kinase inhibitors could be useful for treatment of taxane resistant ovarian tumors.

## Competing interests

The authors declare that they have no competing interests.

## Authors' contributions

CDS performed the flow cytometry, immunofluorescence, drug treatments, and immunoblotting experiments and wrote the initial draft. NL performed the caspase 3/7 and qPCR assays. AOO read and scored the TMA. SL generated the tissue microarray. JFM generated the microarray expression data. BBB provided the ovarian patient tissue samples. CSM directed the research, analyzed the microarray data, and co-wrote the manuscript. All authors read and approved the manuscript.
